# Measuring Single-Cell Phenotypic Growth Heterogeneity Using a Microfluidic Cell Volume Sensor

**DOI:** 10.1038/s41598-018-36000-3

**Published:** 2018-12-13

**Authors:** Wenyang Jing, Brendan Camellato, Ian J. Roney, Mads Kaern, Michel Godin

**Affiliations:** 1 Department of Physics, University of Ottawa, Ottawa, Ontario Canada; 2 Ottawa-Carleton Institute for Biomedical Engineering, University of Ottawa, Ottawa, Ontario Canada; 3 Department of Mechanical Engineering, University of Ottawa, Ottawa, Ontario Canada; 4 Ottawa Institute of Systems Biology, Department of Cellular and Molecular Medicine, University of Ottawa, Ottawa, Ontario Canada

## Abstract

An imaging-integrated microfluidic cell volume sensor was used to evaluate the volumetric growth rate of single cells from a *Saccharomyces cerevisiae* population exhibiting two phenotypic expression states of the *PDR5* gene. This gene grants multidrug resistance by transcribing a membrane transporter capable of pumping out cytotoxic compounds from the cell. Utilizing fluorescent markers, single cells were isolated and trapped, then their growth rates were measured in two on-chip environments: rich media and media dosed with the antibiotic cycloheximide. Approximating growth rates to first-order, we assessed the fitness of individual cells and found that those with low *PDR5* expression had higher fitness in rich media whereas cells with high *PDR5* expression had higher fitness in the presence of the drug. Moreover, the drug dramatically reduced the fitness of cells with low *PDR5* expression but had comparatively minimal impact on the fitness of cells with high *PDR5* expression. Our experiments show the utility of this imaging-integrated microfluidic cell volume sensor for high-resolution, single-cell analysis, as well as its potential application for studies that characterize and compare the fitness and morphology of individual cells from heterogeneous populations under different growth conditions.

## Introduction

Cell biology has traditionally employed the reductionist approach, focusing on the function of individual genes and proteins. But it has become increasingly necessary to understand how cells behave on a systems level as more than the sum of its parts^1,2^. For the cell, phenotypic output can reveal much about the constituents that govern transcription, translation, and signaling. One such phenotypic output is cellular growth rate. Growth rates have been shown to be directly related to gene expression, metabolism, stress response, and the cell cycle^3^. Indeed, precise coordination between growth and the cell cycle is necessary for proper cell division^4^, and its disruption can even lead to carcinogenesis^5^. Yet, continued research efforts are still needed to attain a complete and comprehensive understanding of cell cycle regulation^6,7^. We contend that the development of more precise measurement platforms capable of monitoring cellular growth is crucial in further expanding this research area. Conventional methods often utilize bulk population-level measurements because they are relatively easy and well-established. However, the measurement of population-level averages masks subtleties at the single-cell level^8,9^ where heterogeneity can arise even for clonal populations growing in a homogeneous environment^10,11^. For example, it was only through the measurement single-cell growth rates that Son *et al*.^12^ were able to determine that the cell cycle in some mammalian cells is regulated by a critical growth rate as opposed to a critical cell size. But even when cell cycle is regulated by size, such as in yeast, it can still be coupled to the cellular growth rate^13^. In addition, if mathematical descriptions of cellular function are to be formulated, individual cell data will be needed for precise validation of systems biology models^14^. Chip-based biosensors are uniquely poised to tackle these issues and have seen rising use in single-cell characterization^15,16^, including label-free and non-invasive methods like impedance cytometry^17,18^ and suspended microchannel resonator (SMR) mass sensors^19^.

A crucial factor in understanding cellular function is the strong coupling to environmental conditions, such as salinity^20^, osmotic stress^21^, and nutrient availability^22^. For example, one study has shown that loss of growth control under low nutrient availability can lead to tumorigenesis^23^. *In situ* variation of the growth environment in particular enables experimental evolution at the fundamental cell level and facilitates the study of phenotypic plasticity^24^, for which growth rate can serve as a marker^25^. This of course includes the characterization of drug effects, where individual cell growth and cell cycle data can support models of growth dynamics in response to drug treatment^26^. This is especially important as pathogenic microbes are increasingly evolving resistance to antibiotics^27^ and cancer cells are known to possess mechanisms of multidrug resistance (MDR)^28^. Another environment-dependent evolutionary strategy that can be studied is bet-hedging, where isogenic populations cope with environmental fluctuations by stochastically producing phenotypic heterogeneity with different fitness advantages^29^. A key aspect of bet-hedging is that because the individual cells randomly switch between phenotypes, they inherently generate heterogeneous populations, which can render bulk fitness measurements misleading, as opposed to single-cell measurements.

This paper highlights an imaging-integrated microfluidic cell volume sensor that is able to track growth at the single cell level while controlling the surrounding micro-environment. In this study, we investigate a strain of budding yeast (*Saccharomyces cerevisiae*) exhibiting two phenotypic expression states of the *PDR5* gene, high-expressing (HE) and low-expressing (LE). This gene encodes a transmembrane pump that is part of a MDR network^30^ and is capable of actively removing compounds from the cell^31^. It is one of the best-studied ATP-binding cassette transporters^32^ and is a homologue of the human gene *MDR1* implicated in many drug resistant cancers^33^. Because *PDR5* grants multidrug resistance, the maintenance of a bimodal population could be accompanied by fitness costs.

To characterize the cost/benefit of differential *PDR5* expression, we measured the fitness of individual cells in normal and cytotoxic conditions using a microfluidic cell volume sensor^18^. The device traps an individual cell in a sensing channel and directly measures its volume and growth rate over time. This platform has full microscopy integration and on-chip media exchange, which allows for control of growth conditions and the introduction of drugs. As such, it enables accurate quantification of single-cell reproductive fitness in different environments by measuring cellular volume directly, as opposed to inferring volume by assuming geometries and measuring radii using two-dimensional data acquired by traditional imaging and light scattering methods. Concurrent imaging also enables cell morphology characterization, dynamic visualization of cell division, and the use of fluorescent tags to isolate target cells of interest. We find that the two distinct expression states of *PDR5* do indeed have associated fitness costs depending on the environment, and that the existence of the HE phenotype grants the overall population resilience to potential shifts into toxic conditions. Our results therefore demonstrate the applicability of this microfluidic platform for characterizing the growth response and fitness of single cells in different environments.

## Methods

### Microfluidic Device

**Figure 1:**
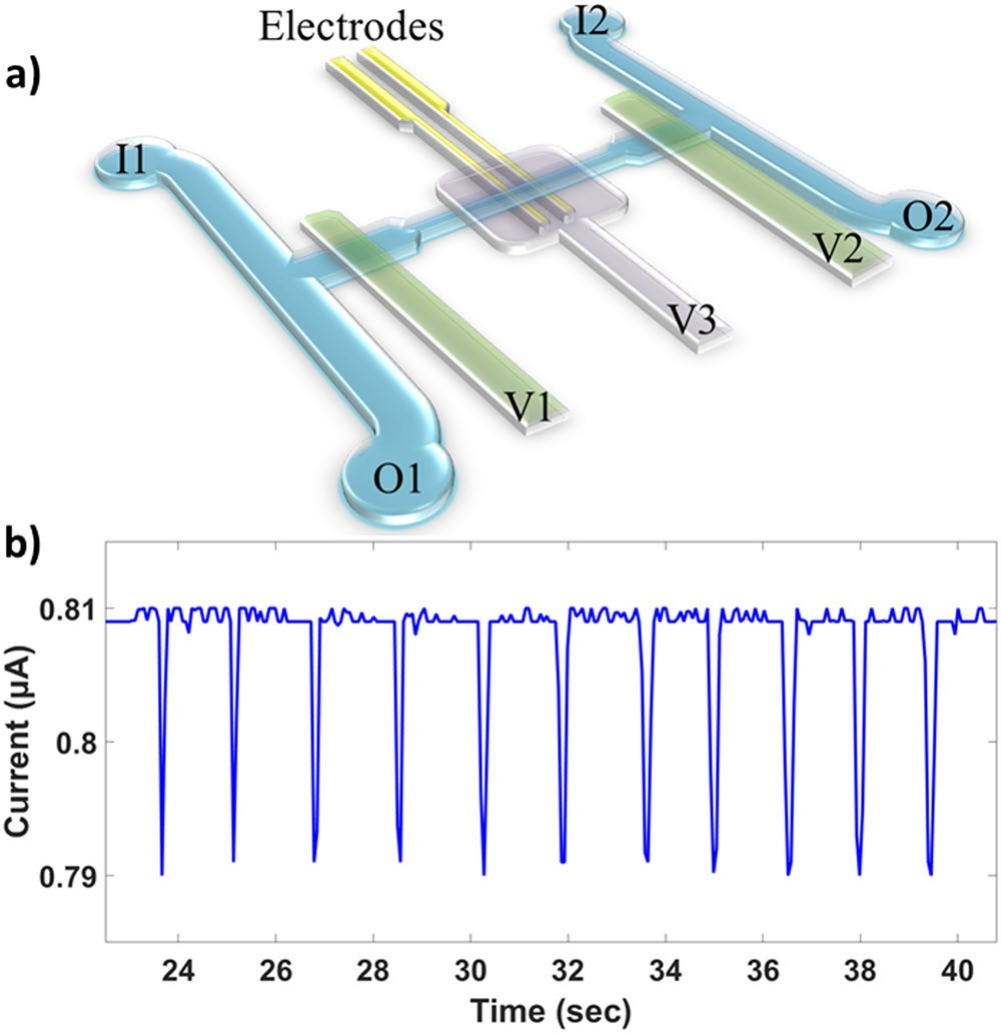
Schematic of the microfluidic device and typical data. (**a**) I1 and I2 denote inlets 1 and 2, both fed from the same vial and pressurized equally. O1 and O2 denote outlets 1 and 2, each empties into its own vial and are pressurized independently. When exchanging the inflowing media to alter the fluid environment, sieve valves V1 and V2 (in green) are used to seal the sensing channel—where the cell is trapped—from the two bypass channels that connect the inlets to the outlets. V1 and V2 are partially lifted during data taking to allow fluid flow whilst preventing the cell from escaping. Sieve valve V3 (in violet) is compressed to decrease the sensing volume and bring the cell closer to the electrodes for enhanced sensitivity^35^. (**b**) This trace of current pulses is an example of data taken for a bead during calibration. A drop in the measured current occurs each time the same particle flows over the electrodes. The amplitude of the drop is proportional to the volume of the cell, which can be tracked over time. Calibrations were performed using the same operating conditions as the experiments (100 kHz, 300 mV amplitude).

The microfluidic cell volume sensor used to quantify volumetric growth in this study was adapted from a similar platform validated previously^18^. The body of the chip is made of elastomeric polydimethylsiloxane (PDMS) fabricated using standard photolithography and multilayered soft lithography^34^. The PDMS body is oxygen plasma-bonded to a piece of glass on which the gold planar electrodes were patterned. Integrated valves allow the deformation of the adjacent fluidic microchannels. As shown in Fig. 1, the current design incorporates an additional compression valve (V3) over the electrodes to provide tunable sensitivity by changing the sensing volume and the maximum distance of a particle from the electrodes as well^35,36^. Metal deposition was done using electron beam evaporation and the electrode geometry was patterned using photolithography. Excess metal was then etched using aqua regia and hydrofluoric acid. Each electrode is 20 μm wide, 50 nm thick with a 5 nm titanium adhesion layer, and their inner edges are 30 μm apart. The sensing channel spanned by the electrodes is 25 μm wide and 20 μm tall when the valve V3 is not compressed.

During experiments, the chip is maintained at 30 °C and mounted on the microscope stage. Bright-field imaging was done in transmission mode where the transparency of PDMS allowed for clear illumination. The sensing electrodes are driven with an AC signal of amplitude 300 mV and frequency 100 kHz. The frequency was chosen so that the effects of the electrode polarization impedance were minimized (see Supplementary Fig. S3) and so that the measurement was only sensitive to cell size and not to the dielectric properties of the cell membrane or cytoplasm^37,38^. The input signal passes through a unity gain low-noise preamplifier before going to the chip. The output signal is converted to a voltage using a transimpedance amplifier and then fed to a lock-in amplifier. When completely sealing the sensing channel, valves V1 and V2 (Fig. 1) are set to a pressure of 35 psi. When they are opened to a partially compressed state, the pressure is set to 24 psi. The valve over the electrodes, V3, is set to 14.5 psi to reduce the channel height for enhanced sensitivity whilst not restricting the passage of cells, including mothers with two attached daughters. The two inlets are fixed at the same pressure while the two outlet pressures are each adjusted on a feedback loop for dynamic control of the pressure gradients in order to cycle the cell over the electrodes. As the cell is shuffled back and forth, the estimated upper limit on the diffusion time for a galactose molecule to reach the cell from the bypass channels is ~2 min, as the real time will be reduced by Taylor-Aris dispersion. Nutrient replenishment on this timescale should be more than sufficient for these growth experiments where even the fastest reproduction time is on the order of 2 hours. In order to correlate the volume of the cell to the amplitude of the current pulses, a calibration was done beforehand using polystyrene microspheres of known size (see Supplementary Fig. S4), where the calibration is kept accurate by dynamic corrections to any baseline drift, which is discussed elsewhere^18^. All data acquisition and the automation of pressure regulators were done via LabVIEW (National Instruments, Austin, TX).

### Yeast Cultures

The base culture media used was yeast extract-peptone-galactose (YPgal), which consists of 2% w/v yeast extract, 4% w/v bacteriological peptone, and 2% w/v galactose, dissolved in DI-H_2_O. For on-chip measurements, 50 mM NaCl and 0.5% w/v bovine serum albumin (BSA) were added to YPgal; this represents the rich media environment in this study. The 50 mM concentration of NaCl provides an optimized ionic current for measurements whilst being low enough to not affect growth. Cycloheximide was added to YPgal with 50 mM NaCl and 0.5% BSA to form the drug environment in this study, with a final cycloheximide concentration of 0.1 µg/mL.

For the low-expressing cells used, initial colonies were prepared by streaking agar plates using cells from a frozen stock that were cultured in YPgal and incubated for up to 24 hours at 30 °C. The low-expressing cell culture was inoculated from one of these colonies, incubated overnight in YPgal, and then diluted once. The sample was taken during log-phase, once the diluted culture reached an OD_600_ of 0.1 to 0.4. For the high-expressing cells, plates were streaked using strictly log-phase cultures (and OD_600_ not exceeding 0.4). Samples of the high-expressing cells used for the on-chip experiments were then taken from log-phase cultures inoculated using only the smallest, slowest-growing colonies, as these colonies were formed from an initial high-expressing cell. All the single cells were randomly chosen from the liquid culture and so were not cell cycle synchronized.

### Tagging PDR5 with GFP

Yeast enhanced green fluorescent protein (yEGFP) was fused to Pdr5 by using homology-based transformation assisted recombination (TAR) cloning to insert a yEGFP coding sequence directly downstream of the *PDR5* gene, along with an 8 amino acid linker to maximize fluorescence. A KanMX cassette was also placed downstream of yEGFP for selection of successful recombinants. The parent BY4741 strain (genotype S288C^a^: *MAT*a; *his*3Δ1; *leu*2Δ0; *met*15Δ0; *ura*3Δ0) was used as a background for the recombination. An area of homology to the C-terminal sequence of *PDR5* was amplified using primers that would exclude the stop codon. Another area of homology upstream of the stop codon was also amplified to ensure efficient integration of the construct in the correct position. The two homologous sequences, as well as the yEGFP coding sequence and the KanMX cassette, were fused to each other in a pairwise fashion using overlap extension PCR. The construct was then transformed into the BY4741 strain.

### Flow Cytometry

Yeast cultures in log-phase growth were diluted 10X in 50 mM sodium citrate buffer. Flow cytometry was performed with a CyAn ADP 9 Analyzer (#CY20130, Beckman Coulter, Inc.) using a 488 nm solid-state laser to excite the yEGFP fluorophore. Fluorescence emission was detected with a 530/40 filter. All Flow Cytometry Standard file formats (.fcs) were analyzed using MATLAB (The Mathworks, Natick, MA) with a custom FCS extraction and analysis script.

## Results

### *PDR5* is Expressed Bimodally in YPgal Cultures During Log-phase Growth

**Figure 2:**
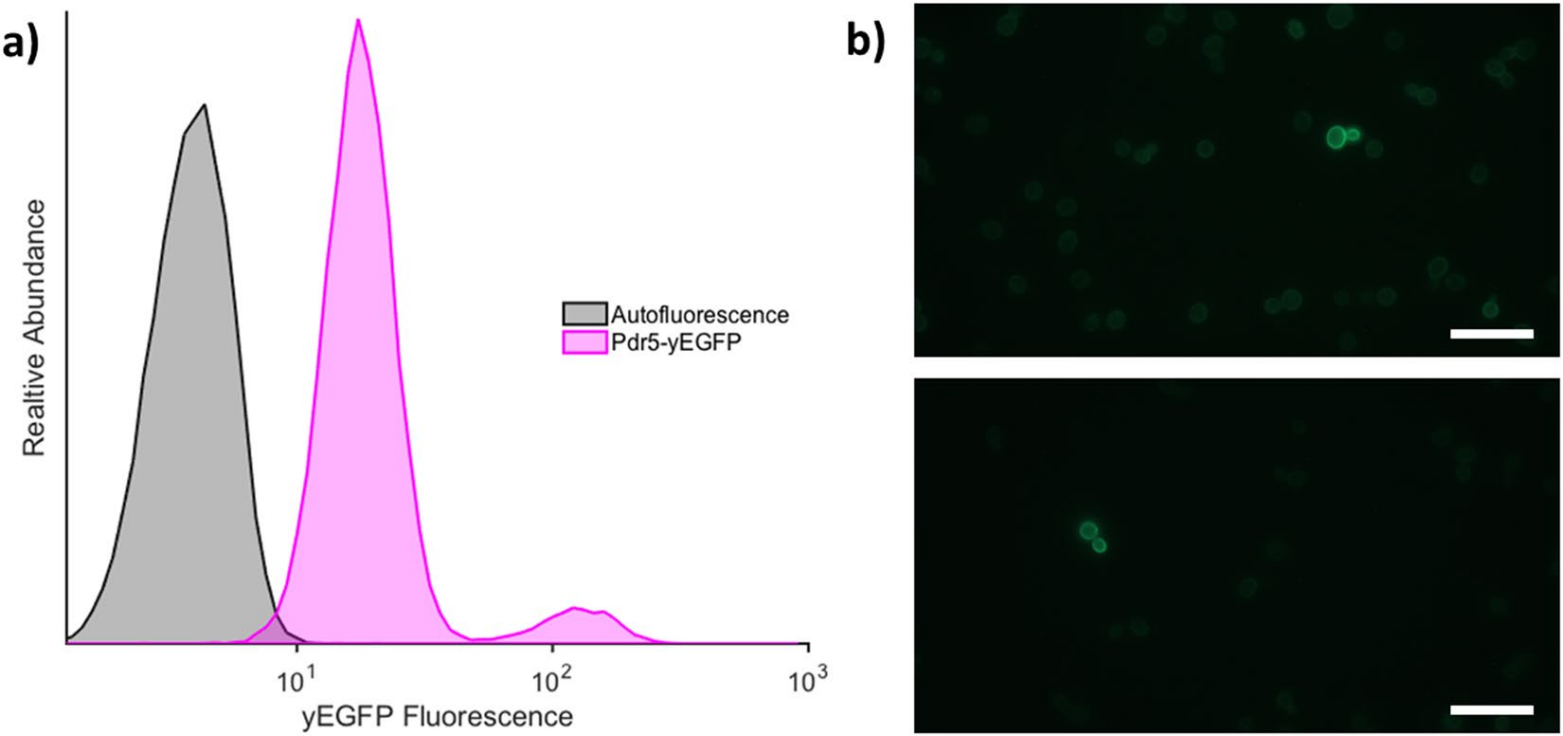
Confirmation of dual phenotypes. (**a**) Tracking of yEGFP-tagged Pdr5 expression. The yEGFP fluorescence from the yEGFP-tagged Pdr5 strain (Pdr5-yEGFP) is compared to the autofluorescence from the wild type strain as measured by flow cytometry. The two peaks of the yEGFP-tagged population show two distinct expression levels of *PDR5*. (**b**) Direct observation of HE cells within the population. Microscope images of yEGFP fluorescence are taken using a 100x objective with a 2 second exposure. Each image shows a single mother-daughter pair of HE cells amongst the LE majority. The bright ring around the border of the cells reflects the concentration of yEGFP at the cell membrane, as expected, since *PDR5* encodes a membrane transporter. The scale bar is the same for both images and is 20 µm.

Tagging Pdr5 with yEGFP successfully allowed us to observe its expression pattern. Figure 2a shows that even the basal fluorescence in the Pdr5-yEGFP strain, correlating with the basal *PDR5* expression, is higher than the auto-fluorescence of wild type cells. Using flow cytometry to characterize the *PDR5* expression, we found that for log-phase cells grown in YPgal, the majority (approximately 94%) exists in a low-expressing (LE) state, while a small subpopulation (approximately 6%) persists in a high-expressing (HE) state with a *PDR5* expression level that is about 10 times higher. This difference in *PDR5* expression is also evident in the fluorescence intensity of the HE cells compared to the surrounding LE cells shown in Fig. 2b. The size of this high-expressing subpopulation is fairly consistent across different cultures in log-phase growth. We therefore show phenotypic heterogeneity of *PDR5* expression in budding yeast.

### The Fitness Advantage of Each Phenotype Depends on the Environment

In order to evaluate the fitness of each phenotype and their corresponding disparities, the aforementioned microfluidic cell volume sensor was used. Cells were flowed into the microfluidic device in the rich media they were cultured in, a target was then trapped in the middle sensing channel and the sieve valves were closed to seal it off from the two bypass flow channels. A fluorescence image was then captured to ascertain the expression level (see Supplementary Fig. S2 for examples). The inflowing fluid was then changed while keeping the trapped cell in the sensing region, to either regular media with 50 mM NaCl as the rich media environment or this same solution spiked with 0.1 μg/mL cycloheximide as the drug environment. The device was thoroughly flushed with the new solution and the valves sealing the central channel were then partially lifted so that the fluid could be shuffled back and forth until the trapped cell was also immersed in the changed media. With automated pressure control, the cell was cycled over the electrodes during the experiment and volume measurements were taken every few seconds. A fluorescence image was also taken at the end of the experiment to evaluate the final expression state.

**Figure 3:**
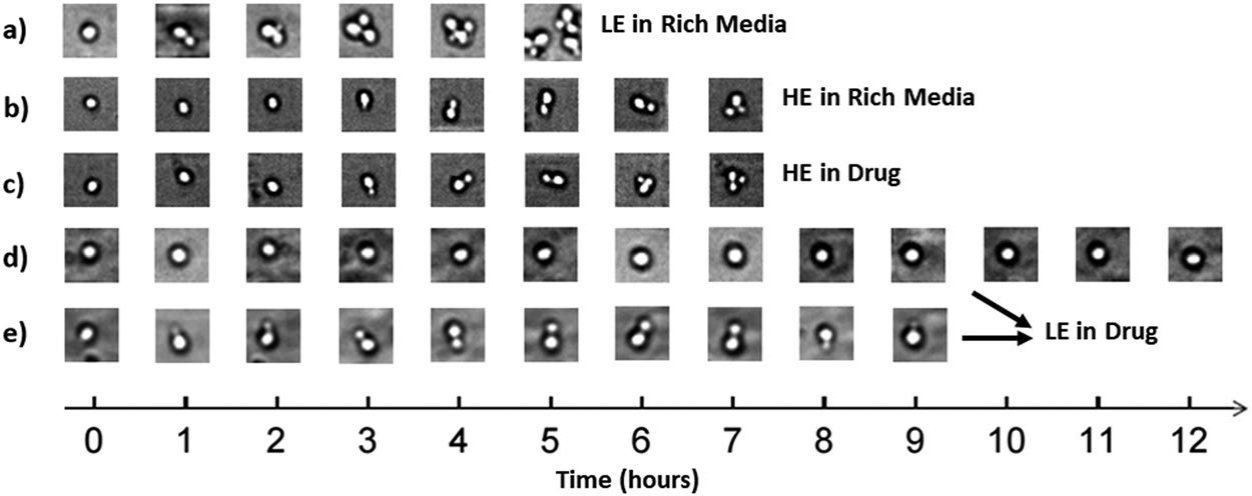
Typical time-lapsed images from the beginning to the end of each respective experiment. (**a**) LE cell in rich media. (**b**) HE cell in rich media. (**c**) HE cell in cycloheximide. (**d**) LE cell in cycloheximide that was not close to division at the start. (**e**) LE cell in cycloheximide that began division at approximately the same time as when cycloheximide was introduced, however shrinking of daughter occurred later, resulting in unsuccessful division.

Concurrently, time-lapsed images of the cells were taken over the course of the experiment to observe the time between budding events. The time between the emergence of the first and second daughters is an approximation of the doubling time and reproductive rate, the means of which are summarized in Table 1. In Fig. 3, sample images for each experimental category provide an outline of the timescales of reproduction. LE cells in rich media display the greatest absolute fitness as depicted in Fig. 3a, reaching a total of 8 cells after 5 hours. The HE cells, while lower in fitness in rich media (Fig. 3b) compared to the LE cells in rich media, are resilient to the shift into a cytotoxic environment, and, as seen in Fig. 3c, growth is not significantly affected. We include two categories for LE cells measured in the drug environment, as shown in Fig. 3d,e, as there were two distinct observations. The first (Fig. 3d) was no division at all within the timeframe of the experiment, and the second (Fig. 3e) was an observed emergence of the first daughter followed by death later on with shrinking of the bud. Both cases show a stark contrast to their growth in rich media (Fig. 3a).

**Table 1:** Averaged data from individual experiments (between 8 and 13 experiments were performed for each of the four conditions, see Supplementary Tables S1–S4 for details).

	LE Cells in Rich Media	HE Cells in Rich Media	LE Cells in Cycloheximide	HE Cells in Cycloheximide
Mean Single-cell Growth Rate × 10^−3^ (μm^3^/sec)	1.5 ± 0.2	0.9 ± 0.1	0.27 ± 0.05	0.85 ± 0.04
Mean Collective Growth Rate with 1 Bud × 10^−3^ (μm^3^/sec)	4.2 ± 0.3	2.7 ± 0.2	1.0 ± 0.1	2.7 ± 0.2
Mean Time Between Appearance of 1^st^ & 2^nd^ Daughters (min)	115 ± 3	180 ± 20	530 ± 50	190 ± 10
Reproductive Success Among All Trials	100%	100%	0%	100%

The uncertainties in the quoted values are the standard errors associated with each computed mean. Reproductive success is characterized by observing at least one successful mitotic division from time-lapsed images. The percentage is with respect to all experiments per condition. T-tests of interest are tabulated in Supplementary Table S5.

**Figure 4:**
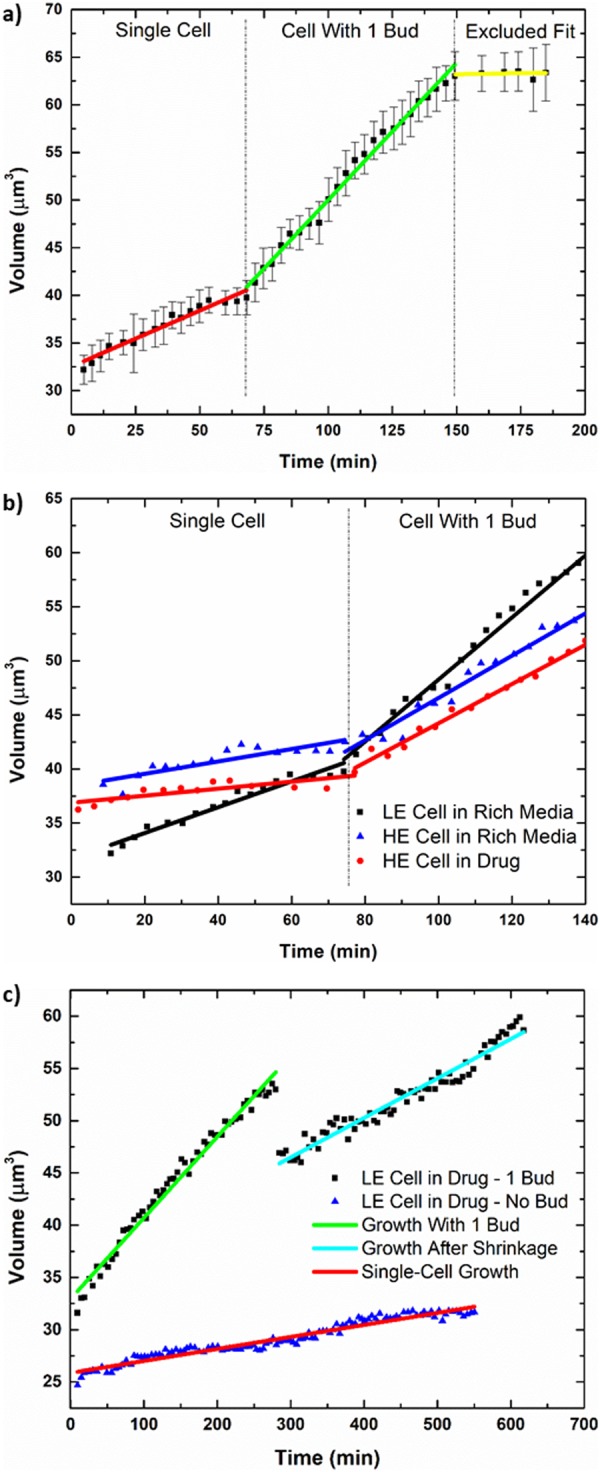
Examples of individual growth curves analyzed. Each data point shown here is a ~5 min average of the raw data points. Error bars represent the standard deviation of the raw data points about this average. (**a**) Example growth curve from an individual experiment of a LE cell in rich media. This shows the typical size of the measurement error bars for the device as well as the separate growth phases, one of which is excluded from consideration. Although growth is known to be exponential, linear fits are applied as a first-order approximation due to the size of the error bars. (**b**) A representative comparison of the growth curves for a LE cell in rich media and HE cells in rich media and in drug. The data points were offset in time to align the emergences of the first bud for better juxtaposition of each growth phase. The typical error bars depicted in (**a**) are not shown and only one curve is included for each case in order to optimize visual clarity. The growth rates exemplified here reflect the results shown in Table 1 as well as the distributions shown in Supplementary Fig. S1. (**c**) Examples of the two cases observed for LE cells in drug. The first curve (green and cyan) is of a LE cell that was able to bud or had begun budding at the start of the experiment but would later experience an abrupt drop in the measured volume (see Fig. 3e). Despite the shrinkage of the first daughter, the mother remains alive and there is still continued growth. Two fits are performed for each phase. The latter rate is not used for statistical comparison purposes since it only occurs for LE cells in drug. The second curve (red) shows the case of a LE cell that was never able to bud and simply continues to increase in size over time.

A representative example of the volumetric growth curves measured is shown in Fig. 4a. The curve is from an individual experiment of a LE cell in rich media and highlights the typical size of measurement error bars as well as the different phases from which growth rates are extracted. The growth rate values for each individual experiment can be found in Supplementary Tables S1–S4 (see Supplementary Information) while the overall averages are listed in Table 1 (normal distributions of the individual growth rates are shown in Supplementary Fig. S1). All tabulated growth rates were quantified from the raw data via analysis using MATLAB. To estimate the growth rates, we used linear regression to compute the slopes, as the size of the error bars obscures subtler temporal changes in volume. In addition, the initial size of single cells is also estimated using the intercept from the linear regression of only the single-cell growth phase (these values are also tabulated in Supplementary Tables S1–S4). We note that although cellular volume during growth is known to increase exponentially^19^, the first-order approximation captures the discernable trends in the growth curve given measurement uncertainties. One of the slopes measured is the volumetric growth rate for the single-cell phase (red line in Fig. 4a). For some trials, the mother began with a daughter or produced one shortly thereafter, thus leaving an insufficient amount of data for the single-cell phase. For this reason, we also include a growth rate corresponding to just after the emergence of the first daughter, delineated as ‘Collective Growth Rate with 1 Bud’ (see Table 1 and Supplementary Information). The starting point for this region coincides approximately with when the time-lapse images show the emergence of the first daughter. The rate is extracted from the fit shown by the green line in Fig. 4a and represents the growth rate leading towards mitosis from just after entry into the S phase, when the collective growth rate is measured to be the greatest. Since cycloheximide is a eukaryotic translation inhibitor, this rapid growth stage should be particularly sensitive to it. Following this growth is a negative change in the slope, as shown by the yellow fit in Fig. 4a, despite there still being only two cells. This is due to the deceleration in growth rate as the mother proceeds through mitosis and back into the G1 phase, and it is excluded from data consideration. As shown in Table 1, the mean collective growth rates compare with one another in a fashion consistent with how the mean single-cell growth rates compare with one another. Figure 4b shows representative growth curves of a LE cell in rich media (black) together with HE cells in rich media (blue) and in drug (red) as a comparative example of their growth rates, while Fig. 4c shows examples of the two cases observed for LE cells in drug. Error bars here are of the same magnitude as those shown in Fig. 4a but are not shown for Fig. 4b,c for greater visual clarity of the superimposed data. As can be seen in Fig. 4b, the LE cell in rich media has the greatest slopes, as expected, and the HE cells show very similar growth rates in both rich media and the drug environment. This agrees with the results in Table 1 and is confirmed by the statistical analysis discussed later.

For Fig. 4c, the data points in black exemplifies the case for LE cells that had begun budding at the onset of the experiment or shortly thereafter, and the data points in blue shows the case for LE cells that were able to grow in size but were inhibited from successful initiation of cell division due to their low *PDR5* expression (also shown in Fig. 3d,e, respectively). For the cells already engaged in cell division, we observe that after a period of growth, the daughter abruptly shrinks and continues to do so over time while the mother continues to grow. Growth following this drop is shown by the cyan line (Fig. 4c) and the associated linear fit was performed for the cases where this was observed (for data, see Supplementary Table S4).

In comparing the tabulated averages, we see that in the rich media environment, LE cells not only grow faster than the HE cells but also have the highest absolute growth rates and the shortest time between divisions. P-values comparing the growth rates of LE cells in rich media to those of HE cells in rich media are all less than α = 0.01 (p-values of 0.0027 and 0.0002 for single-cell growth rate and collective growth rate with 1 bud, respectively. See Supplementary Table S5 for all t-tests of interest and p-values). Thus, the LE phenotype has the optimum fitness in this condition and the highest absolute fitness overall. In the toxic environment we see that the mean single-cell growth rate for HE cells is very similar to the corresponding rate in rich media and are within experimental uncertainty with a p-value of 0.6244. In addition, the mean collective growth rate with 1 bud is the same for HE cells in both rich media and in cycloheximide (p-value of 0.8776). The interdivision times are also similar, with a p-value of 0.5998. Thus, there is no statistically significant difference between the fitness of HE cells in rich media vs those in drug.

LE cells measured in cycloheximide, however, have the worst fitness and the greatest disparity between environments. Not only are the growth rates the lowest amongst all four regimes, there is a fivefold drop from their mean single-cell growth rate in rich media. The LE cells also experienced a unique phenomenon whereby those that began division shortly before or after the start of the experiment—when drugged media was switched into the device—were unable to successfully complete reproduction with the first daughter despite continued growth, and always led to the shrinking of the daughter cell. While not shown in Table 1, there was continued overall growth even after this event, as was shown in Fig. 4c. For the cases in which this was observed, the growth rate following the abrupt drop in measured volume was determined and the mean of all such values was computed to be 0.6 ± 0.1 × 10^−3^ μm^3^/sec. It is less than the mean collective growth rate with 1 bud, which represents the procession towards mitosis, but it is greater than the mean single-cell growth rate, which represents the sole growth of the mother cell. There were three cases where a second daughter was able to emerge near the end of the experiment despite the apparent death of the first, allowing for estimates of the interdivision time. However, cells that were not close to division at the beginning of the experiment were greatly inhibited from doing so. While these single cells did continue to grow, they did not attempt to initiate division over an experimental time period of at least 4 times the duration required for reproduction by LE cells in rich media. Therefore, LE cells in cycloheximide either attempted division and failed or were prevented from doing so altogether, resulting in 0% reproductive success over the course of the experiment. These factors only serve to exacerbate the fitness of the LE phenotype in the drug environment.

## Discussion

Using single-cell growth measurements, we were able to show that different *PDR5* expression phenotypes confer different fitness advantages depending on the environment. In the absence of drug, we found the LE subpopulation to indeed be more fit than the HE subpopulation due to faster growth rates and faster reproduction. We note here that although it is known that larger cells grow faster than smaller ones^19^, the estimates for the initial single-cell sizes showed no statistically significant difference amongst all four experimental conditions (see Supplementary Table S6 for p-values), and so we contend that initial size is not a determining factor for differences in the observed growth rates. As stated earlier, two-tailed t-tests comparing the mean single-cell growth rate of LE cells to HE cells in rich media, as well as the mean collective growth rate with 1 bud of LE cells to HE cells in rich media, both yielded a statistically significant difference. However, the rarer HE subpopulation’s slower growth is a tradeoff for increased resilience to a toxic environment, where there was no statistically significant difference between the fitness of the HE cells in rich media vs in drug. The fitness disparity between LE cells in the two environments, however, is drastic, with a 5-fold drop in the mean single-cell growth rate and a 4-fold drop in the mean collective growth rate with 1 bud when in the drug environment, as shown in Table 1. T-tests comparing the mean single-cell growth rate of the LE cells in drug to those of the HE cells in both environments were also statistically significant (p-values of 0.0001 or less). This confirms that the growth rate of LE cells in drug is indeed the lowest. Therefore, the relative fitness of each phenotype is environment dependent, with LE cells performing better in rich media and HE cells performing better in drug. In addition, the LE cells experienced failed attempts at reproduction or complete inhibition of it. Cells in the other three regimes were all able to demonstrate successful advancement through mitosis; LE cells in cycloheximide were the only ones where 0% of the samples had reproductive success. This further establishes the poor fitness of this phenotype in the drug environment. It is evident that although there is a fitness cost associated with having a slower growing subpopulation under the ideal growth conditions of rich media, these HE cells are still able to thrive under toxic conditions, thus conferring an environment dependent fitness advantage to the population as a whole.

We note that although these observations are for the particular concentration of cycloheximide chosen in this study, additional environmental variations are entirely implementable if a study requires it. We chose the cycloheximide concentration of 0.1 μg/mL for the experiments to ensure an effect on cellular growth without completely inhibiting it (including for the LE cells) so that growth rates could be evaluated. Other studies with budding yeast have used concentrations up to three orders of magnitude higher^39–41^, and it has been shown that lower concentrations do not completely prevent protein synthesis^42^. In fact, a study which used 0.1 μg/mL demonstrated that protein synthesis rates can stabilize at a lower constant value^43^. Therefore, it is likely that at this concentration, the effect of cycloheximide is simply small for the HE cells, as the mean single-cell growth rates showed no statistically significant difference. While it would be interesting to see what the ceiling of tolerance would be for the HE cells before their fitness in the drug environment begins to wane, we deemed it unnecessary for showing that there is in fact an environment dependent fitness disparity between the two phenotypes and that there is also a benefit to paying the fitness cost for maintaining a bimodal population in an ideal growth environment.

The triggers and mechanisms underlying the shrinking event observed for LE cells in drug remain unclear. Cycloheximide is a broad inhibiter of protein synthesis in eukaryotes, thus it should interfere with all phases of the cell cycle. However, some of the LE cells were able to begin division shortly after introduction of the cycloheximide-dosed media. This implies that despite a toxic presence, they successfully proceeded into S phase. This next phase is not immediately aborted and is maintained for up to several hours until an abrupt shrinking event occurs over a period of seconds. Though the effects of cycloheximide could be playing a role in impeding and delaying apoptosis^40^, how a necrotic death is prompted only after prolonged exposure is unclear. As for why there is an abrupt drop in the measured volume, we note that at the relatively low driving frequency of 100 kHz, an intact cell membrane contributes a large capacitive impedance^44^, but a sudden loss of membrane integrity would result in an increase of ionic current through the volume occupied by the cell and a corresponding decrease in the magnitude of the current pulse. This is supported by the fact that necrotic death is characterized by a rapid increase in the permeability of the plasma membrane^45^. Thus, this platform allows for rapid detection of changes to the dielectric properties of the cell membrane as well as the characterization of subtle changes to membrane integrity over time, which can be useful for studying drugs that target membrane permeability. However, the dielectric properties of cell death are better characterized by impedance spectroscopy^46^. We also observed that the mean growth rate following the shrinking event is greater than the mean single-cell growth rate of the LE cells in drug while being less than their mean collective growth rate before the shrinking occurred. For this, it is understandable that cell contents would leak into the environment given the loss of membrane integrity, but considering the comparison that was just given, it may be possible that the mother was also reabsorbing some of the matter in the bud.

For the LE cells that did not initiate division at the beginning, they were never observed to do so throughout the entirety of the experiment. It is most likely that the insufficient number of efflux pumps resulted in a concentration of cycloheximide within the cell that was able to keep it arrested in the G1 phase^42^. The G1 checkpoint can be effectively obstructed by blocking the translation of important proteins for the transition into S phase^47,48^. But in three of the cases where division had been initiated at the beginning of the experiment, a second bud was observed emerging later on despite the previous death of the first daughter. However, their viabilities were undetermined. Since the presence of cycloheximide induces increased *PDR5* transcription^49^, it was possible that those mother cells were able to produce more efflux pumps over time so as to expel enough cycloheximide and manage a degree of protein synthesis that allowed for procession through the G1 checkpoint. However, none of this culminated in confirmed reproductive success over the observation period.

The evidence presented here demonstrates that each of the two phenotypes confers a different reproductive fitness advantage depending on the environment. Thus, by maintaining a small subpopulation of high *PDR5* expression cells, the entire population avoids the risk of complete extinction in the event of a sudden shift to a highly toxic environment where greater resistance is needed. However, it is not entirely clear if the over-expression of *PDR5* is the sole cause of the slower growth, though it could be a contributing factor as excessive protein production taxes cellular resources. In addition, as *PDR5* encodes a transmembrane efflux pump, it may also lead to indiscriminate removal of nutrients or other necessary compounds from within the cytosol. This pump also requires ATP hydrolysis to function, thus an over-abundant number of them would become an onerous energy expenditure for the cell. In fact, a recent study found that in *S. cerevisiae*, excessive expression of proteins containing membrane-protruding regions resulted in a very high fitness cost^50^. But this coupling of high *PDR5* expression to slower growth is perhaps to be expected as it is consistent with studies that have shown slowed growth is correlated to stress resistance and response^3,51,52^.

It is worth noting that although the observed fitness disparity is required by the evolutionary strategy of bet-hedging, there are also additional criteria that need to be met^29^, and the mechanisms which control and give rise to the observed phenotypic heterogeneity in *PDR5* expression still remain unclear. While the yeast used is of a single strain, it would be necessary to determine that the high *PDR5* expression is not due to unexpected chromosomal and/or mitochondrial mutations. Given that stochastic processes underlie bet-hedging^53^, random switching between the phenotypic states would also need to be observed. Nonetheless, the fitness requirement of bet-hedging was successfully quantified using this platform, and is therefore fully applicable to such studies. This method also offers the advantage of being able to check the phenotypic state of single cells at any stage via fluorescence, and since bet-hedging individuals inherently switch states, bulk measurements could be misleading about the true nature of such phenotypes.

Using an imaging-integrated microfluidic cell volume sensor capable of measuring single-cell growth rates, we showed that two phenotypic expression states of the *PDR5* gene exhibited by a *S. cerevisiae* population have associated environment-dependent fitness disparities. Through control of the on-chip environment, we found that while the low-expressing cells had higher fitness in rich media, severe deficiencies were apparent in the presence of cycloheximide. The high-expressing cells, however, were able to cope equally well in both conditions, and had higher relative fitness in the drug environment. While we measured fitness as a phenotypic output of gene expression, these results underscore the importance of growth rate as a biophysical marker for cells as well as its potential to serve as a parameter for probing the systems level effects of engineered perturbations. The interplay between cell growth, the cell cycle, and environmental conditions is complex and interconnected, and we hope the applicability of our device for studying these relationships from the single-cell perspective will help to unravel these complexities.

## Electronic supplementary material

Supplementary Information

## Data Availability

The datasets generated during and/or analyzed during the current study are available from the corresponding author on reasonable request.
