# Supplementary material for: Measuring Single-Cell Phenotypic Growth Heterogeneity Using a Microfluidic Cell Volume Sensor
Source: Sci Rep. 2018 Dec 13;8:17809. doi: 10.1038/s41598-018-36000-3 (PMC6293012; doi:10.1038/s41598-018-36000-3)
Supplement: Supplementary file 1 — Supplementary Information [file 41598_2018_36000_MOESM1_ESM.docx]

**Supplementary Information**

Title: **Measuring Single-Cell Phenotypic Growth Heterogeneity Using a Microfluidic Cell Volume Sensor**

Authors: **Wenyang Jing, Brendan Camellato, Ian J. Roney, Mads Kaern, and Michel Godin**

**Supplementary Tables**

In the trials where a single mother cell did not produce a bud during the experiment, began with a bud already, or where the second daughter did not emerge over the course of the experiment, the associated entry in the table is filled with not applicable (NA). The base data was measured in seconds so the following tables show the analysis of the data in its original dimensions. The error on each tabulated growth rate is the error of the slope coefficient from the linear regression. Similarly, the estimates of initial single-cell size are the intercepts from the pertinent fit. Errors on the mean values—quoted in the captions for each of the following tables and tabulated in Table 1—are the standard errors of the mean computed from the tabulated values in each respective column, as shown below.

**Supplementary Table S1. LE Cells Grown On-chip in Rich Media**

| Growth Rate of Single Cell x 10^-3^ (μm^3^/sec) | Collective Growth Rate with 1 Bud x 10^-3^ (μm^3^/sec) | Initial Single-Cell Size (μm^3^) | Time Between Emergence of 1^st^ and 2^nd^ buds (sec) |
| --- | --- | --- | --- |
| 2.0 ± 0.1 | 4.8 ± 0.1 | 32.4 ± 0.3 | 6700 ± 300 |
| 1.96 ± 0.06 | 5.0 ± 0.2 | 24.6 ± 0.2 | 6300 ± 300 |
| 1.2± 0.1 | 3.8 ± 0.2 | 30.5 ± 0.2 | 6700 ± 300 |
| 1.24 ± 0.09 | 4.5 ± 0.2 | 32.6 ± 0.2 | 7500 ± 300 |
| 1.69 ± 0.04 | 5.8 ± 0.5 | 26.6 ± 0.2 | 6700 ± 300 |
| 0.98 ± 0.06 | 3.47 ± 0.07 | 28.8 ± 0.1 | 6500 ± 300 |
| 1.71 ± 0.07 | 3.3 ± 0.2 | 29.0 ± 0.1 | 7200 ± 300 |
| 1.27 ± 0.03 | 3.72 ± 0.06 | 21.1 ± 0.2 | >8400 |
| 1.00 ± 0.06 | 3.5 ± 0.1 | 27.6 ± 0.2 | 7350 ± 450* |
| 2.4 ± 0.2 | 4.2 ± 0.2 | 26.9 ± 0.3 | >5900 |

**Table S1.** The mean growth rates (x 10^-3^) for columns one and two are **1.5 ± 0.2 μm^3^/sec** and **4.2 ± 0 .3 μm^3^/sec** respectively. The mean initial single-cell size from column three is **28 ± 1 μm^3^**. The mean doubling time from column four is **6900 ± 200 sec**. Lower-bounded times indicate that a second daughter did not emerge before the conclusion of the experiment, though it would have occurred given more time. *For this case, a close estimate of the time is unknown, however the upper and lower bounds are known: 6900 < t < 7800. A mean of these bounds is used.

Supplementary Table S2. HE Cells Grown On-chip in Rich Media

| Growth Rate of Single Cell x 10^-3^ (μm^3^/sec) | Collective Growth Rate with 1 Bud x 10^-3^ (μm^3^/sec) | Initial Single-Cell Size (μm^3^) | Time Between Emergence of 1^st^ and 2^nd^ buds (sec) |
| --- | --- | --- | --- |
| 0.76 ± 0.05 | 2.3 ± 0.1 | 27.4 ± 0.2 | 8700 ± 300 |
| 0.9 ± 0.1 | 2.1 ± 0.1 | 36.2 ± 0.2 | 9000 ± 300 |
| 0.59 ± 0.02 | 3.2 ± 0.1 | 25.3 ± 0.3 | 10500 ± 300 |
| 1.0 ± 0.1 | 3.3 ± 0.2 | 38.6 ± 0.4 | 9600 ± 300 |
| 0.92 ± 0.02 | 2.39 ± 0.03 | 21.0 ± 0.2 | 17400 ± 300 |
| 0.73 ± 0.03 | 2.09 ± 0.07 | 24.5 ± 0.2 | 11900 ± 300 |
| 0.8 ± 0.1 | 3.0 ± 0.2 | 35.6 ± 0.2 | 9100 ± 300 |
| 1.5 ± 0.3 | 3.2 ± 0.1 | 35.0 ± 0.6 | 9600 ± 300 |

Table S2. The mean growth rates (x 10^-3^) for columns one and two are 0.9 ± 0.1 μm^3^/sec and 2.7±0.2 μm^3^/sec respectively. The mean initial single-cell size from column three is 30 ± 2 μm^3^. The mean doubling time from column four is 11000 ± 1000 sec.

Supplementary Table S3. HE Cells Grown On-chip in 0.1 μg/mL Cycloheximide

| Growth Rate of Single Cell x 10^-3^ (μm^3^/sec) | Collective Growth Rate with 1 Bud x 10^-3^ (μm^3^/sec) | Initial Single-Cell Size (μm^3^) | Time Between Emergence of 1^st^ and 2^nd^ buds (sec) |
| --- | --- | --- | --- |
| 0.89 ± 0.01 | 3.05 ± 0.08 | 22.5 ± 0.2 | 11000 ± 300 |
| 0.7 ± 0.1 | 2.60 ± 0.08 | 36.4 ± 0.4 | 12800 ± 300 |
| 0.96 ± 0.01 | 2.7 ± 0.1 | 22.4 ± 0.1 | 9700 ± 300 |
| 0.96 ± 0.01 | 3.0 ± 0.1 | 24.5 ± 0.1 | 9200 ± 300 |
| 1.00 ± 0.02 | 3.15 ± 0.09 | 27.9 ± 0.2 | 9800 ± 300 |
| 0.74 ± 0.03 | 2.9 ± 0.2 | 29.0 ± 0.2 | 9900 ± 300 |
| 0.83 ± 0.02 | 2.3 ± 0.1 | 27.0 ± 0.2 | 14500 ± 300 |
| 0.69 ± 0.07 | 1.55 ± 0.04 | 26.8 ± 0.4 | 14400 ± 300 |

Table S3. The mean growth rates (x 10^-3^) for columns one and two are 0.85 ± 0.04 μm^3^/sec and 2.7 ± 0.2 μm^3^/sec respectively. The mean initial single-cell size from column three is 27 ± 2 μm^3^. The mean doubling time from column four is 11400 ± 800 sec.

Supplementary Table S4. LE Cells Grown On-chip in 0.1 μg/mL Cycloheximide

| Growth Rate of Single Cell x 10^-3^ (μm^3^/sec) | Collective Growth Rate with 1 Bud x 10^-3^ (μm^3^/sec) | Initial Single-Cell Size (μm^3^) | Growth Rate After Shrinkage x 10^-3^ (μm^3^/sec) | Time Between Emergence of 1^st^ and 2^nd^ buds (sec) |
| --- | --- | --- | --- | --- |
| NA | 1.40 ± 0.03 | NA | 0.87 ± 0.04 | 27000 ± 300 |
| 0.50 ± 0.01* | NA | 25.6 ± 0.1 | NA | NA |
| NA | 0.83 ± 0.02 | NA | 0.35 ± 0.07 | 36600 ± 600 |
| NA | 0.862 ± 0.006 | NA | 0.8 ± 0.2 | >50300 |
| NA | 0.81 ± 0.02 | NA | NA (observed at end) | >28000 |
| NA | 1.29 ± 0.02 | NA | 0.60 ± 0.02 | 32400 ± 600 |
| 0.127 ± 0.003** | NA | 20.88 ± 0.06 | NA | NA |
| NA | 0.82 ± 0.03 | NA | 0.47 ± 0.02 | >33000 |
| 0.431 ± 0.006* | NA | 25.7 ± 0.1 | NA | NA |
| 0.207 ± 0.005*** | NA | 28.9 ± 0.2 | NA | NA |
| 0.159 ± 0.004*** | NA | 24.4 ± 0.2 | NA | NA |
| 0.192 ± 0.004*** | NA | 25.86 ± 0.09 | NA | NA |
| 0.241 ± 0.006*** | NA | 32.6 ± 0.1 | NA | NA |

**Table S4.** The mean growth rates (x 10^-3^) for columns one and two are **0.27 ± 0.05 μm^3^/sec** and **1.0 ± 0.1 μm^3^/sec** respectively. The mean initial single-cell size from column three is **26 ± 1 μm^3^**. For the case where shrinking occurred, the mean growth rate (x 10^-3^) from column four is **0.6 ± 0.1 μm^3^/sec**. The mean doubling time from column five is **32000 ± 3000 sec**. For some of the trials where there was only single-cell growth, the cells experienced an initial transient state of faster growth before reaching a prolonged steady state of slower growth during which no successful division was ever observed. The fit for this latter steady state phase is used as the single-cell growth rate reported above in column 1 as it more accurately reflects the cell’s stabilized growth rate in the drugged environment. However, the intercept from the fit for the initial transient state is used to estimate the initial single-cell size. However, no such values are reported for the trials where the single-cell growth rates are NA as those cells had already begun initiating division. Some doubling times are lower-bounded because no second daughter had emerged by the end of the experiment. *No budding was observed for experimental durations of 10 and 11 hours respectively. **No budding was observed for an experimental duration of 21 hours. ***No budding was observed for an experimental duration of 9 hours.

Supplementary Table S5. Two-Tailed T-Tests for Growth Rates Assuming Unequal Variances

| Null Hypothesis | t-statistic | degrees of freedom | p-value | α |
| --- | --- | --- | --- | --- |
| Mean single-cell growth rate for HE cells in drug *equals* mean single-cell growth rate for HE cells in rich media | 0.5052 | ≈10 | 0.6244 | 0.01 |
| Mean growth rate with 1 bud for HE cells in drug *equals* mean collective growth rate with 1 bud for HE cells in rich media | 0.1569 | ≈14 | 0.8776 | 0.01 |
| Mean doubling time of HE cells in drug *equals* mean doubling time of HE cells in rich media | 0.5378 | ≈13 | 0.5998 | 0.01 |
| Mean single-cell growth rate for LE cells in rich media *equals* mean single-cell growth rate for HE cells in rich media | 3.5893 | ≈15 | 0.0027 | 0.01 |
| Mean collective growth rate with 1 bud for LE cells in rich media *equals* mean collective growth rate with 1 bud for HE cells in rich media | 4.7832 | ≈15 | 0.0002 | 0.01 |
| Mean single-cell growth rate for LE cells in drug *equals* mean single-cell growth rate for HE cells in rich media | 5.7233 | ≈11 | 0.0001 | 0.01 |
| Mean single-cell growth rate for LE cells in drug *equals* mean single-cell growth rate for HE cells in drug | 8.3352 | ≈12 | <0.0001 | 0.01 |

Supplementary Table S6. Two-Tailed T-Tests for Initial Single-Cell Size Assuming Unequal Variances

| Null Hypothesis | t-statistic | degrees of freedom | p-value | α |
| --- | --- | --- | --- | --- |
| Mean initial single-cell size for LE cells in rich media *equals* mean initial single-cell size for HE cells in rich media | 0.9413 | ≈10 | 0.3687 | 0.01 |
| Mean initial single-cell size for LE cells in rich media *equals* mean initial single-cell size for HE cells in drug | 0.4895 | ≈13 | 0.6326 | 0.01 |
| Mean initial single-cell size for LE cells in rich media *equals* mean initial single-cell size for LE cells in drug | 0.9766 | ≈13 | 0.3466 | 0.01 |
| Mean initial single-cell size for HE cells in rich media *equals* mean initial single-cell size for HE cells in drug | 1.1975 | ≈12 | 0.2542 | 0.01 |
| Mean initial single-cell size for HE cells in rich media *equals* mean initial single-cell size for LE cells in drug | 1.5337 | ≈11 | 0.1533 | 0.01 |
| Mean initial single-cell size for HE cells in drug *equals* mean initial single-cell size for LE cells in drug | 0.3731 | ≈13 | 0.7151 | 0.01 |

**Supplementary Figures**


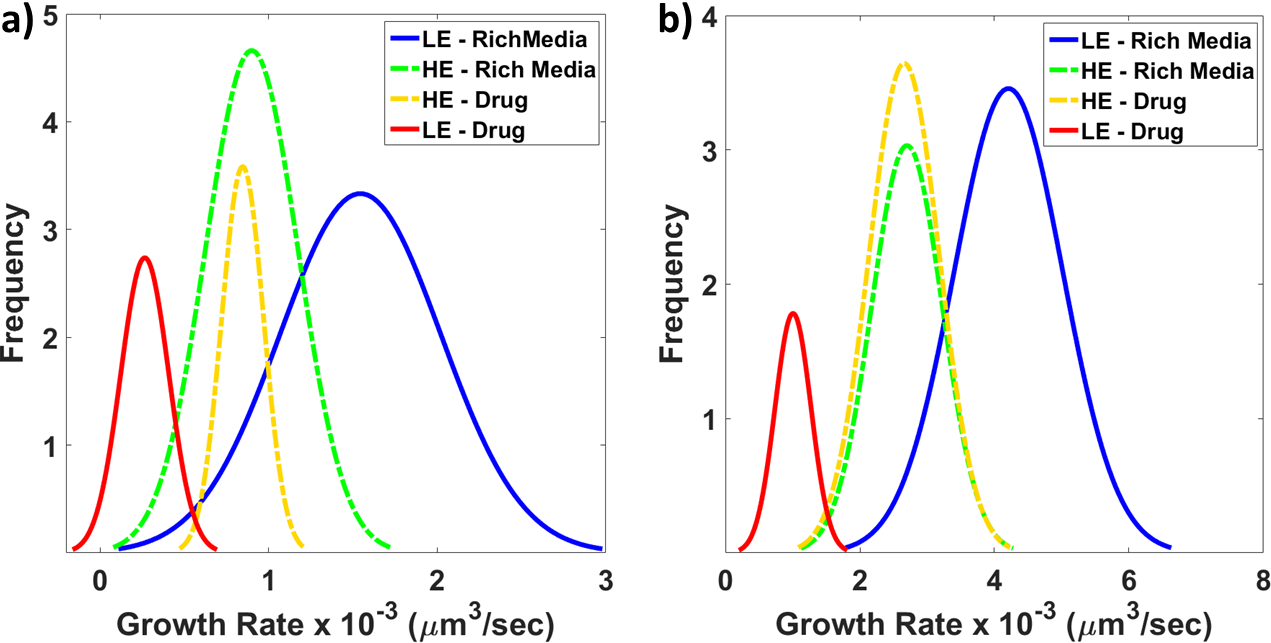


Supplementary Figure S1. These plots show normal distributions fitted to the growth rate data shown in Supplementary Tables S1-S4. This graphical depiction also reflects the results in Supplementary Table S5. (a) Distributions for the single-cell growth rates, or column 1 of Supplementary Tables S1-S4. (b) Distributions for the collective growth rates with 1 bud, or column 2 of Supplementary Tables S1-S4.


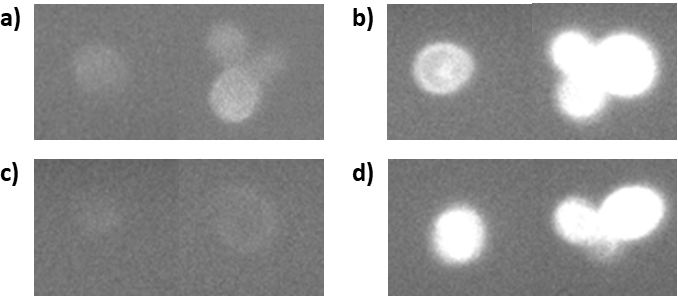


Supplementary Figure S2. These show sample images of the GFP fluorescence intensity taken at the beginning and the end of their respective experiments. These were done to ascertain the phenotype of the cell at the start (left image in each pair) and to see if that state changed at the end (right image in each pair). The brightness and contrast settings are the same for all images (analyzed in ImageJ). (a) LE cells tested in regular media. (b) HE cells tested in regular media. (c) LE cells tested in cycloheximide. (d) HE cells tested in cycloheximide.


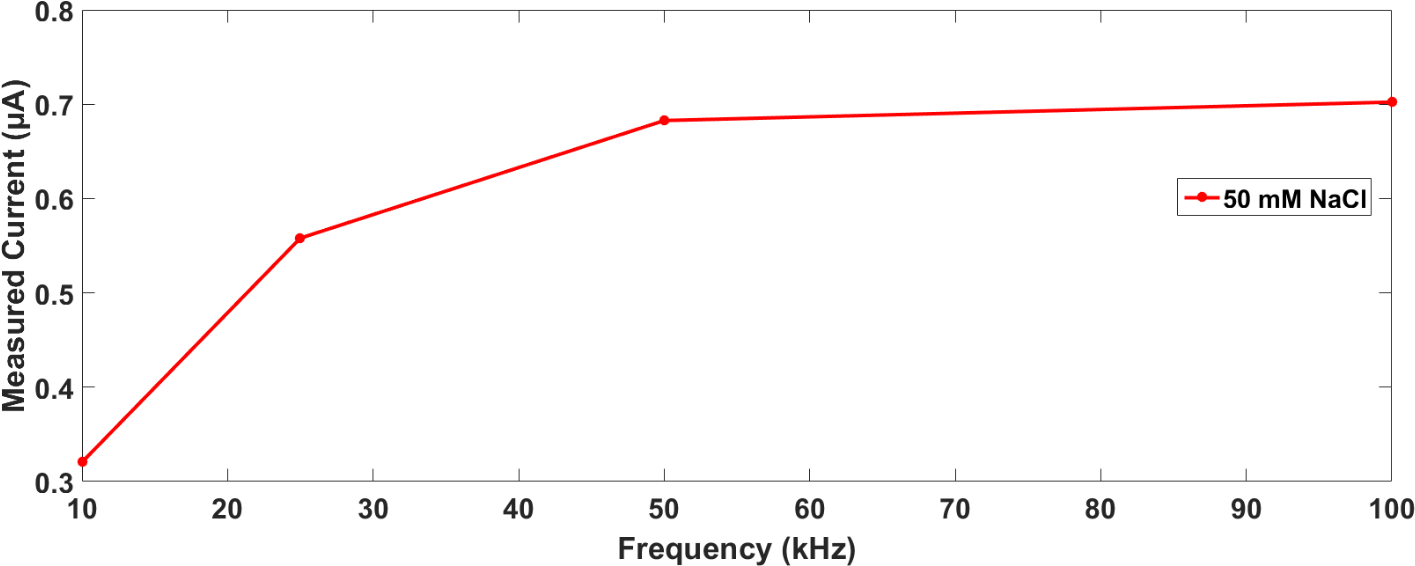


**Supplementary Figure S3.** This shows the baseline current measured by one device in 50 mM NaCl at 300 mV amplitude. Note that as the frequency of the applied signal increases, the measured current increases, which is consistent with reduced electrode polarization impedance. The values of the currents at 50 kHz and 100 kHz are 0.6827 µA and 0.7022 µA, respectively. Thus, it appears that at this salt concentration, minimalization of electrode polarization plateaus at 100 kHz.


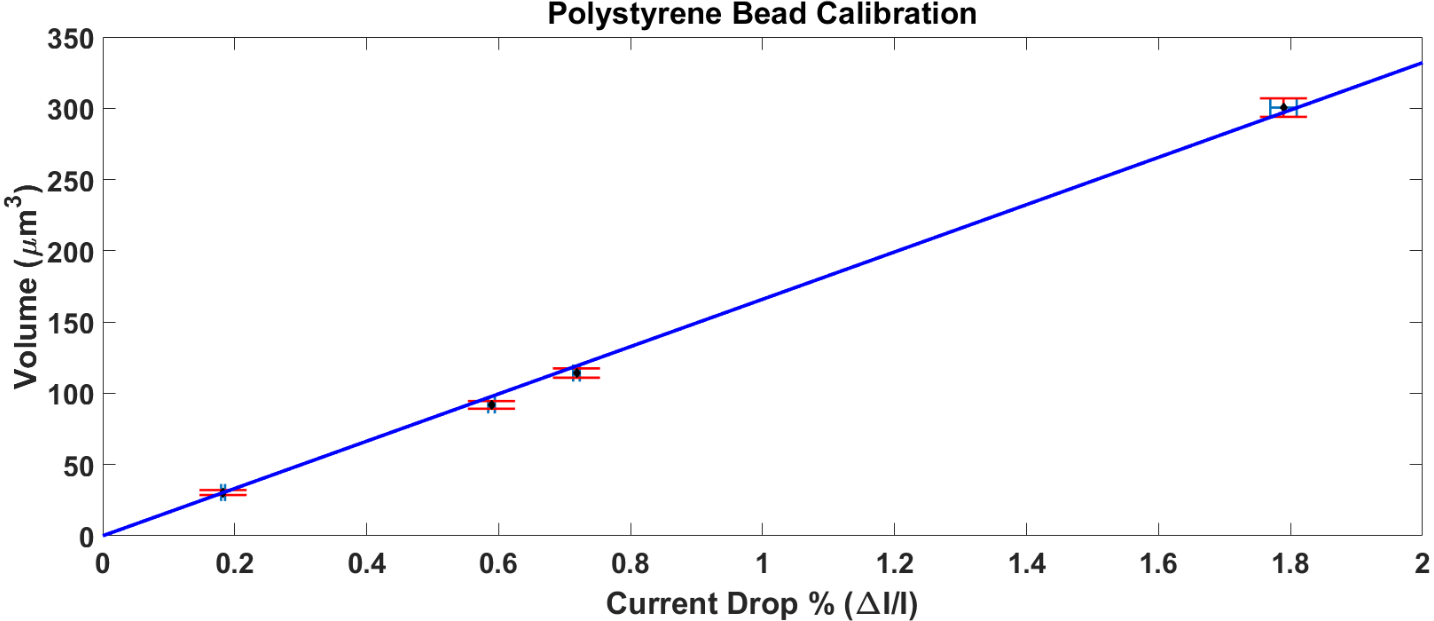


**Supplementary Figure S4.** This shows an example of a calibration performed during this study to translate electrical measurements into volume. When an object passes across the electrodes, the measured current drops. The size of this drop is quantified as a percent change and is proportional to the volume of the object. The calibration is done by measuring these current pulses for polystyrene beads of known sizes (3.81 μm, 5.67 μm, 6.02 μm, and 8.31 μm diameters). As shown above, the relation is linear within our operating conditions. The linear fit (in blue) provides the means of calculating volume from our current pulse measurements. Individual calibrations must be performed based on the device, the solution used, and/or the settings of the electronics.


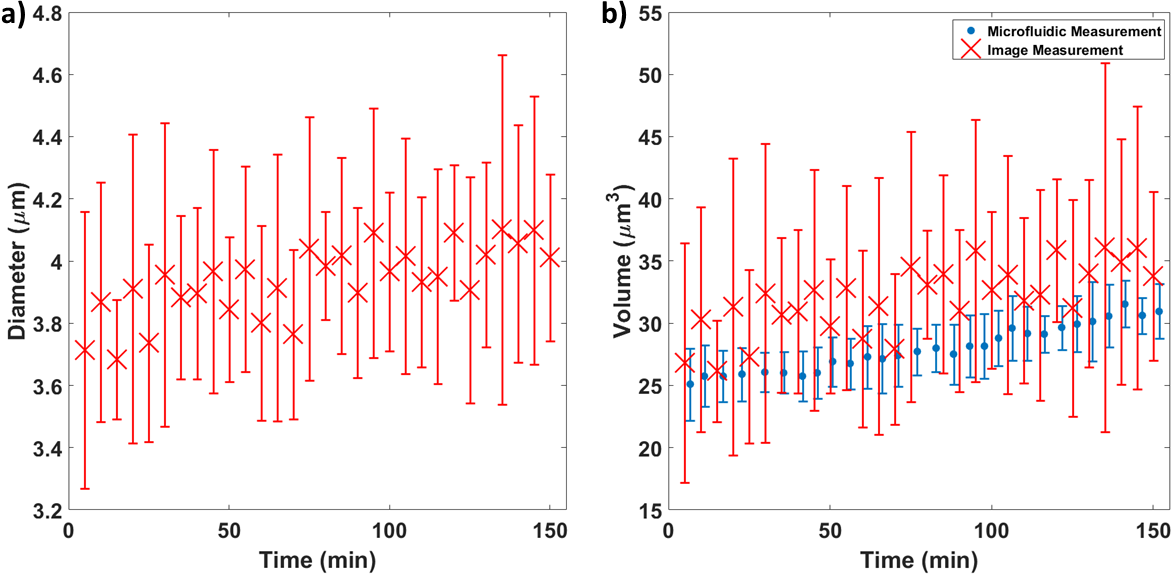


**Supplementary Figure S5.** This shows a comparison of the volume measurements made using the microfluidic sensor to what is calculated from diameter measurements made based on the time-lapsed images taken during the same experiment. It is the HE cell shown in Fig. 3b. Each image-based data point plotted is the average of measurements for every 5 minutes (error bars are standard deviations), where a time-lapsed image was taken every 30 seconds. The data presented is for approximately 2.5 hours of growth in order to establish a comparison for the single-cell phase, where assuming a spherical geometry could be acceptable as during the collective growth phase, the mother and daughter cells become more non-spherical and the daughter is also at times obscured from view due to changing orientations. In addition, the cells themselves can drift in and out of focus (focusing plane is also different in the region compressed by the central valve). **(a)** The diameters were measured in ImageJ. Unsurprisingly, the error bars on the diameter measurements are quite large. However, even accounting for the error bars, the diameters measured this way are consistent with the expected mean single-cell diameter for haploid yeast, which is approximately 4 µm (the strain studied in this work is haploid). Note that estimates for the mean initial single-cell sizes are also given in the captions of Supplementary Tables S1-S4. As this is an HE cell grown in rich media, the mean initial volume given is 30 ± 2 μm^3^, translating to a mean initial diameter of 3.86 ± 0.09 μm, which is consistent with expectations. **(b)** The volumes for the image measurements are calculated using the diameter estimates whilst assuming a spherical geometry. As can be seen, the image measurement yields significantly larger error. Consequently, the precision is worse compared to the microfluidic device. Thus, the image-derived volumes are not in disagreement with the microfluidic volume measurements.
